# Ultra-Processed Profits: The Political Economy of Countering the Global Spread of Ultra-Processed Foods – A Synthesis Review on the Market and Political Practices of Transnational Food Corporations and Strategic Public Health Responses

**DOI:** 10.34172/ijhpm.2021.45

**Published:** 2021-05-24

**Authors:** Rob Moodie, Elizabeth Bennett, Edwin Jit Leung Kwong, Thiago M. Santos, Liza Pratiwi, Joanna Williams, Phillip Baker

**Affiliations:** ^1^Melbourne School of Population and Global Health, University of Melbourne, Melbourne, VIC, Australia.; ^2^College of Public Health, Medical and Veterinary Sciences, James Cook University, Townsville, QLD, Australia.; ^3^International Center for Equity in Health, Federal University of Pelotas, Pelotas, Brazil.; ^4^Indonesian Adolescent Health Association, Jakarta, Indonesia.; ^5^Institute for Physical Activity and Nutrition, Deakin University, Geelong, VIC, Australia.

**Keywords:** Ultra-Processed Foods, Sugar-Sweetened Beverages, Corporations, Corporate Power, Advocacy

## Abstract

**Background:** Ultra-processed food (UPF) and Ultra-processed beverage (UPB) consumption is associated with higher risks of numerous non-communicable diseases (NCDs). Yet global consumption of these products is rising due to profound changes in production, processing, manufacturing, marketing, retail, and consumption practices, alongside the growth of the resources and political influence of Big Food. Whilst the sales of UPFs and UPBs in high-income countries (HICs) are stagnating, sales are rapidly expanding in more populous middle-income countries (MICs). In this paper, we adopt a political economy of food systems approach to understand how growth of Big Food in MICs drives the NCD pandemic.

**Methods:** We conducted a mixed methods synthesis review. This involved quantitative data collection and development of descriptive statistics; a search for academic, market and grey literature on the expansion of UPF in MICs; and the development of themes, three illustrative case examples (South Africa, Colombia, and Indonesia), and synthesis of the enablers of successful campaigns in MICs into recommendations for public health campaigns.

**Results:** We project that the combined sales volume of UPFs in MICs will reach equivalency with HICs by 2024, and the total sales volume of UPBs in MICs is already significantly higher than in HICs. Similarly, annual growth in UPF sales is higher in MICs compared to HICs. We also show how Big Food has entrenched its presence within MICs through establishing global production and hyper-local distribution networks, scaling up its marketing, challenging government policies and scientific expertise, and co-opting civil society. We argue that public health can counter the influence of Big Food by developing an expanded global network of driven and passionate people with diverse skillsets, and advocating for increased government leadership.

**Conclusion:** The projected increase in sales of UPFs and UPBs in MICs raises major concerns about the global capacity to prevent and treat NCDs.

## Background

The commercial determinants of health (CDOH) are the “strategies and approaches used by the private sector to promote products and choices that are detrimental to health.”^1^ CDOH are receiving growing attention from researchers, advocates, and policy-makers with the purpose of monitoring and informing societal responses to ‘manufactured’ epidemics.^2,3^ The rising burden of non-communicable diseases (NCDs) is amongst the most significant pandemics of our time, as NCDs are the leading cause of death and disability worldwide.^4^

Historically, research on the prevention of NCDs has focused on metabolic and behavioural risk factors rather than upstream determinants, such as the production, marketing, and distribution of ultra-processed foods (UPFs), and the power of the industry that produces them. In this paper, we adopt a political economy approach that is increasingly being used to understand how growth of the UPF industry drives the current pandemics of obesity and diet-related NCDs.^3,5-9^ This relationship is informed by a rapidly growing body of evidence which shows that UPF consumption is associated with poorer diet quality, and higher risks of obesity, cardiovascular disease, type 2 diabetes, certain cancers, depression, and all-cause mortality.^10-12^ Greater production and intensive marketing of UPF products are also generating significant environmental degradation, including plastic waste entering marine ecosystems.^13,14^

We adopt the definition of UPFs as “products with additives and industrially processed ingredients that have been technologically broken down and modified.”^15^ Examples include sugar-sweetened beverages (SSBs), confectionery, savoury snacks, refined baked goods, sweetened yoghurts, biscuits, and many varieties of fast food. We use UPF to refer to both UPFs and beverages, although we distinguish between these categories where relevant by referring to ultra-processed beverages (UPBs).

In this paper, we examine the role of transnational UPF corporations (which we refer to as ‘Big Food’) as a vector of disease through the production, marketing, distribution and political activity of promoting these products on a global scale.^1,2,16-19^ We focus on the industry’s expansion into low- and middle-income countries (LMICs) where nearly 80% of NCD-related deaths occur, and related morbidity is rapidly increasing.^20,21^ MICs are more likely than high-income countries (HICs) to be affected by the double burden of malnutrition, food insecurity, and under-nutrition, as well as an increase in obesity and related complications.^19^ Policies limiting the consumption of UPF products are fundamental to efforts to combat NCDs in LMICs.^22^ However, there is increasing evidence that the market and political practices of Big Food shape patterns of health and disease, and pose a risk to the development and implementation of effective NCD prevention policies.^3,7,26-28^

 In this paper, we address questions requiring much greater attention in the public health literature. What explains the rapid growth in the size and global reach of the UPF industry in MICs? How do these transnational corporations (TNCs) then sustain these high consumption levels? To answer these questions, we examine this global expansion within its historical context and the growing power of TNCs to shape food systems on a global scale. We examine the political and market strategies used by Big Food to expand in MICs, including efforts to undermine effective public health regulations in three countries – South Africa, Colombia, and Indonesia. These MICs were chosen using a convenience sample, based on access to existing research and journalistic reporting, to show the diversity of growth strategies used in three regions which have seen significant investment from Big Food over the last two decades. Our analysis explores these countries’ experience as MICs over the past decade, with the understanding that their status as ‘emerging markets’ for Big Food may change as they transition to becoming HICs. We then propose some recommendations that public health campaigns should adopt to limit the corporate power of Big Food in MICs. We conclude by considering what the projected expansion of Big Food in MICs over the next decade means for NCD prevention and treatment.

## Key Messages

**Implications for policy makers**

- Middle-income countries (MICs) now represent huge markets for Big Food and are likely to grow significantly over the next decade.
- The projected expansion of Big Food and ultra-processed food (UPF) markets in MICs raises major concerns about the global capacity to prevent and treat non-communicable diseases (NCDs).
- To effectively respond to the NCDs pandemic, governments must understand the market and political practices used by Big Food to establish, grow, and sustain its markets in MICs.
- We propose recommendations that public health campaigns should include to effectively monitor and counter these market and political practices.
- Governments, civil society groups and public health practitioners need to work together to build long-term global networks and social movements with diverse skillsets to mitigate the harms associated with Big Food.

**Implications for public**

UPF consumption is associated with higher risks of obesity, cardiovascular disease, type 2 diabetes, certain cancers, and other non-communicable diseases (NCDs). Yet consumption of these products is rising worldwide. This reflects profound food system changes currently underway – including production, processing, manufacturing, marketing, retail, and consumption – alongside growth in the size, resources and global reach of Big Food. As the sales of UPFs in high-income countries (HICs) are stagnating, sales are rapidly expanding in MICs. Using new data, we project that the combined sales in MICs will outweigh sales in HICs by 2024 and demonstrate the increasing importance of MICs for Big Food’s growth. We also show how Big Food uses a corporate playbook of market and political practices to establish, grow and sustain its markets. This expansion raises major concerns about the capacity of MICs to prevent and treat NCDs.

## Methods

Given the complexity of the topic, we adopted a mixed methods synthesis review method that draws from diverse data sources. This involved quantitative data collection and development of descriptive statistics; a search for academic, market, and grey literature; and development of themes, illustrative case examples, and synthesis of results. Each step was guided by the growing number of CDOH frameworks which identify and categorise the strategies used by corporations.^2,17,18,29-36^

###  Quantitative Data Collection and Analysis

Market share data (percentage of market sales attributed to a global company) were sourced from the Euromonitor Passport Database for the world’s largest 80 markets for the years 2011-2019. Market sales volume data (kg) were sourced from the same database for the years 2006-2019, with projections to 2024, for UPF and UPB categories. The methods used by Euromonitor to collect this data are described elsewhere.^6,37^ Sales volume data has been used in similar analyses in other studies^6,9^ and were converted to a per capita basis using population estimates from the World Bank World Development Indicators Database. Countries were categorised by the World Bank income categories. Descriptive statistics and figures were generated using R version 4.0.2.

###  Search for Relevant Literature 

 We searched academic databases (Google Scholar, Scopus, Web of Science, EconLit, MEDLINE, Embase, PsycINFO, Business Source Premier, and CINAHL), industry news sources, and the websites of international organisations and non-governmental organisations (NGOs). We first searched for literature published since 2006 using a combination of product (ultra-processed foods*, ultra-processed beverages*, sugar-sweetened beverages*) and industry (industry*, corporations*, company*, market*, commercial*) related terms identified from the CDOH frameworks. We then searched for articles examining the political economy of Big Food, using the same search terms as the first search strategy along with the terms such as ‘policy,’ ‘lobbying’ and ‘politics.’ To identify additional grey literature, these search strategies were supplemented by hand searches of references lists.

###  Development of Themes, Case Examples and Synthesis of Results

 Included literature was reviewed to identify key themes relevant to the aim of the study. To illustrate the market and political practices used by industry to promote consumption of UPFs, we included three country case examples. To develop these examples, we searched Google, industry news sources and government documents using a combination of product, industry, and corporate power (economy, partnership, support, donate, investment, consumption) related terms. We also consulted with experts working in the field. Searches were conducted in English and the national language of each country (Spanish and Bahasa Indonesia). We then studied the social media accounts and websites of relevant Big Food TNCs and civil society organisations to contextualise the corporate practices. Finally, we synthesised the data and literature to create recommendations for public health campaigns.

## Results

 The results are divided into three sections. First, we describe global trends and dynamics in UPF markets. Second, we summarise the market and political practices used by TNCs to establish, promote, and maintain high levels of UPF consumption within MICs. Finally, we present three country case examples – South Africa, Colombia, and Indonesia – to illustrate how Big Food has established, grown and protected its markets.

###  Global Trends and Dynamics in UPF Markets 

Market sales data from 2006-2019 have shown that per capita UPF consumption has reached remarkably high levels in HICs, with levels significantly higher than in upper-middle income countries (UMICs) and LMICs.^6^ Survey data show that UPFs contributed 42%, of dietary energy intake in Australia in 2011-2012^38^ and 58% in the US in 2009-2010.^39^ The contribution of UPFs to dietary energy intake is currently much lower in UMICs and LMICs than in HICs, ranging from 21.5% in Brazil in 2008-2009^41^ to 29.8% in Mexico in 2012.^42^ However, whilst growth is relatively stagnant in HICs, UPF market sales, and the contribution of these products to energy intake are rapidly growing in UMICs and LMICs.^43^

**Figure 1:**
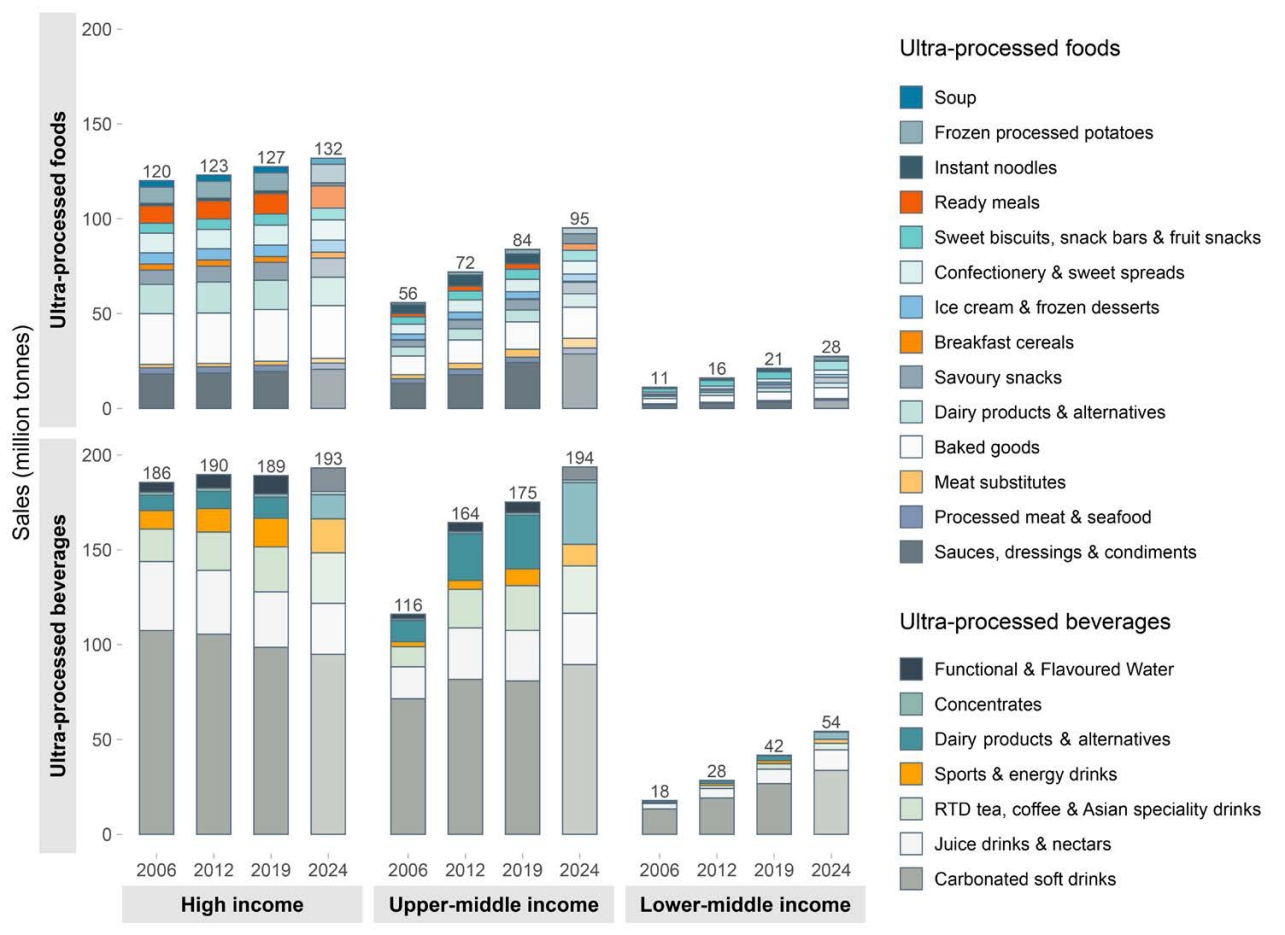
Total Market Sales Volumes of UPF and UPB, 2006-2019 With Projections to 2024, by Country Income Level. Abbreviations: UPF, ultra-processed food; UPB, ultra-processed beverage.

**Figure 2:**
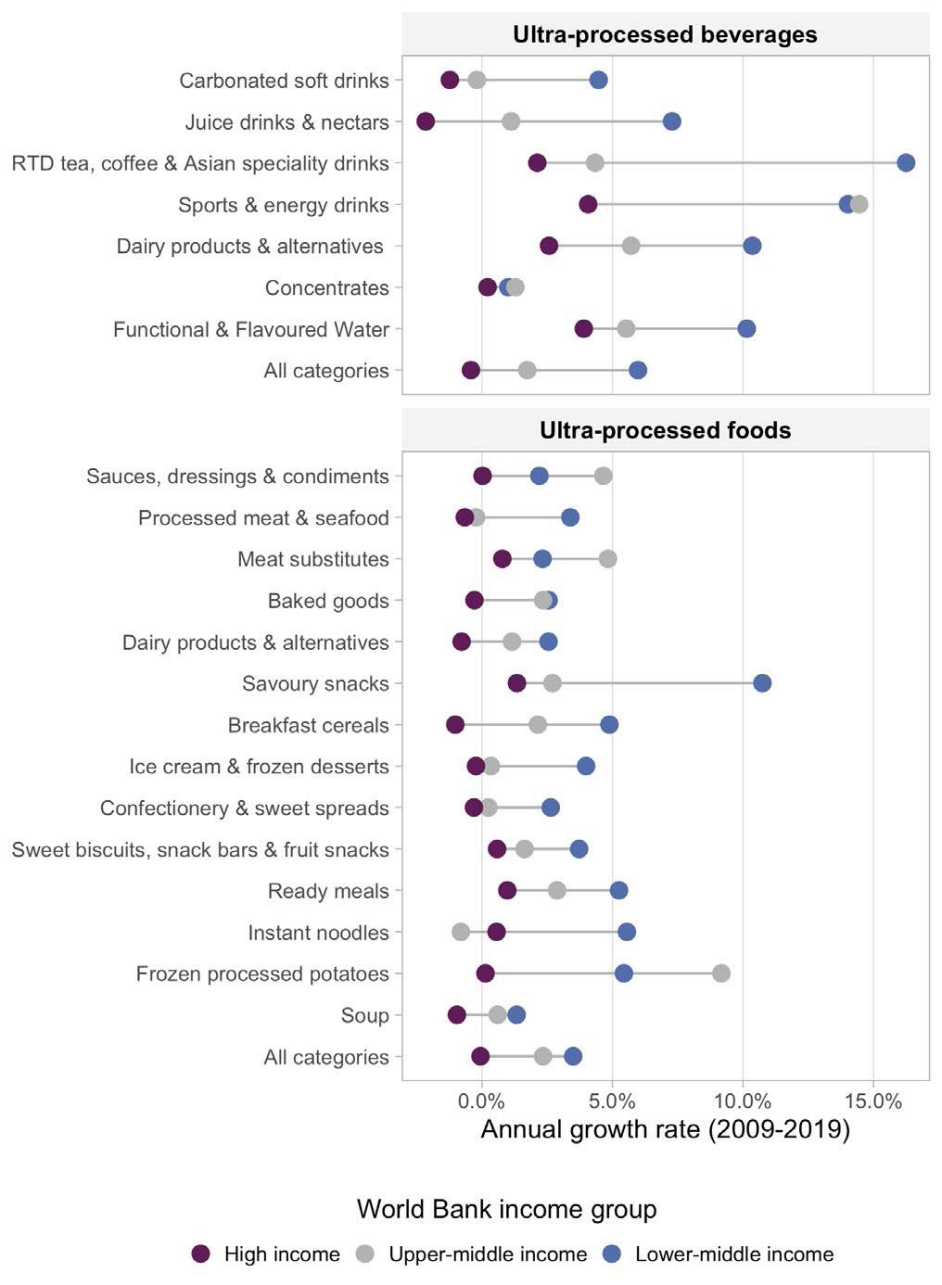
Annual Average Growth Rate (%) in Per Capita UPF and UPB Sales, for the 2009-2019 Period, by Country Income Level. Abbreviations: UPF, ultra-processed food; UPB, ultra-processed beverage.

We present new data from 2019, shown in Figure 1, which projects that the combined sales volume of UPFs in UMICs and LMICs will reach equivalency with HICs by 2024, and the total sales volume of UPBs in UMICs and LMICs is already higher than in HICs. Additionally, as shown in Figure 2, annual growth in UPF sales is much higher in LMICs (3.5%) and UMICs (2.3%) compared to HICs (-0.1%). Similarly, annual growth in UPBs in LMICs (6.0%) and UMICs (1.7%) is much higher than in HICs where markets are shrinking (-0.4%). This data indicates that UMICs and LMICs are now as important as HICs to Big Food in terms of market size, and more important in terms of growth. As markets in HICs begin to stagnate, Big Food is moving to pursue growth opportunities in UMICs and LMICs, attracted by their large, growing and increasingly urbanised populations whose incomes are rising.^6^

###  Strategies Used to Establish, Promote, and Sustain High Levels of UPF Consumption 

Whilst there have been many different categorisations of the corporate playbook used to establish and promote UPF consumption,^17,18,29-36,44^ we broadly categorised these strategies into market practices and political practices. These practices are defined as applied business strategies and tactics employed to advance a corporation’s economic performance and create a more favourable external environment.^45^

####  Market Practices 

 We identified three main categories of corporate market practices used to grow and sustain UPF markets: establishing global production networks, establishing large-scale and hyper-local distribution networks, and scaling up marketing.

####  Big Food’s Transnational Expansion – Establishing Global Production Networks

The first strategy for establishing and growing UPF markets is the transnational expansion of corporations through the establishment of globally integrated sourcing and production networks. This expansion is enabled by TNCs’ access to finance that facilitates their growth, vast human resource capabilities and knowledge capital, trademarks and global brand recognition, logistical and manufacturing technologies, and capacity to adapt operational practices to diverse regulatory, economic and social contexts.^43,46,47^

Big Food includes some of the leading corporations of economic globalisation. The industry’s expansion rapidly accelerated in the 1980s as domestic markets in North America and Europe became increasingly saturated, and LMICs became more open to foreign trade and investment via rapid industrialisation and income growth.^48^ The establishment of the World Trade Organization in 1995, and the subsequent increase in regional and bilateral trade agreements, supported corporations to move investments, production inputs, and final products across borders, expand their intellectual property protections, and foster market deregulation.^49,50^

The rapid growth in the flow of foreign direct investment from corporations headquartered in HICs into MICs demonstrates where TNCs intend to expand in the long-term.^49^ This takes the form of investments in new production capacity through greenfield investments in manufacturing plants, distribution centres, and research and development units; mergers with, or acquisitions of, domestic competitors; and the expansion of networks of franchisees and affiliated partners. These investments have made the productive capacities of Big Food extensive. For example, the Coca-Cola system includes 225 bottling partners and 900 bottling plants, generating 2 billion servings sold every day in over 200 countries.^51^ The McDonald’s system has 38695 outlets in 119 countries, most of them owned and operated by franchisees.^52^

In many instances, rapid growth is achieved through partnership with or acquisition of domestic competitors. For example, Coca-Cola became the market leader in India in 1993 by acquiring Parle Products’ leading soft drinks brands, including Thums Up Cola.^53^ Similarly, Nestlé acquired the Chinese companies Hsu Fu Chi and Yinlu in 2011 to tap into growing Chinese demand for UPFs.^54^ These acquisitions include tangible productive assets of domestic corporations as well as intangible assets, such as staff expertise and knowledge of local market conditions and cultural preferences, existing relationships with suppliers, and pre-established distribution networks.^49^

The increased capacity of TNCs to shift investments, manufacturing plants, and jobs internationally translates into significant political power as governments compete for these investments. This can include governments deregulating their markets or providing tax or other concessions for those corporations. The power of TNCs grows as their investments and the market for their products increase within a country. This is because their impact on the labour market, knowledge transfer to domestic firms, and purchasing of domestic production inputs become increasingly important to the country’s economy.^18^ With increased power and leverage over governments, TNCs can more effectively avoid or reduce payment of corporate tax.^55^ This in turn reduces the capacity of the government to finance health services and programmes,^55^ and the public health system’s capacity to prevent and treat NCDs.

####  Big Food’s Sub- national Expansion – Establishing Hyper-local Distribution Networks

 Big Food’s expanding global sourcing and production networks function as the main vectors for the spread of UPFs across countries. However, sophisticated distribution strategies are used to make UPF products widely available to consumers across many different market segments and localities within these countries.

The growth of supermarkets and convenience stores is a significant driver of the nutrition transition in many MICs.^43^ Although supermarkets can have positive impacts on food safety and improve nutrition in some circumstances,^56^ they also act as a major distribution channel for UPFs and processed foods. Due to the economies of scale in the high-volume supply chains of TNCs and chain supermarkets’ procurement contracts, supermarkets can provide UPFs at a much lower per unit cost than traditional retailers.^6^ For example, in Brazil the share of UPFs as a proportion of total food purchased was 25% higher at supermarkets and prices for these products 37% lower compared to other food retailers.^57^

Where modern supermarkets do not exist, Big Food uses hyper-localised distribution strategies to reach poorer and rural populations at ‘the base’ of the consumer pyramid. For example, Coca-Cola provides store-owners the goods necessary to run tiendas, which are informal vendors or family-run general stores, on the condition that the tienda stock and promote Cola-Cola’s drinks in Mexico.^50^ Similarly, it was reported in 2010 that the Nestlé até Você micro-distribution system uses 7000 door-to-door saleswomen to sell Nestlé’s ‘affordable nutrition’ products to 250000 households in Brazilian favelas.^58,59^ These types of employment programmes reinforce the economic dependence of countries on TNCs.

####  Marketing and Promotion Practices

Big Food fosters and sustains sales growth by employing an integrated, pluralistic, and rapidly evolving range of marketing techniques aimed to increase the consumption of its products by local consumers. The World Health Organization (WHO) defines marketing as “any form of commercial communication or message that is designed to, or has the effect of, increasing the recognition, appeal and/or consumption of particular products and services.”^60^ This section will only focus on digital marketing and corporate social responsibility (CSR) programmes as they are increasingly being used to expand markets in MICs.

Digital marketing has facilitated the marketing of unhealthy foods to become more targeted, personalised, and capable of changing consumer behaviour. It is designed to spread rapidly (‘virally’) on the internet, and can be separated into three types of content: paid (eg, targeted/personalised ads and influencer endorsements), user-generated (eg, content generated by users – including shares, likes and comments) and owned (eg, brand-owned websites, apps, and social media platforms). These types of content are often used in integrated approaches, and further enhance the ‘glocalisation’ strategies used by Big Food, where corporations adapt their products and marketing to local cultures of consumption and regulatory contexts. These strategies have proven very successful at increasing consumption, with data from Europe demonstrating that combining online marketing with marketing on television and in cinemas can amplify returns on investment by approximately 70%.^61^

Big Food is considered to be at the forefront of innovation within digital marketing.^62^ A major part of the success of Big Food’s digital marketing strategy is spurred by what Shoshana Zuboff describes as ‘surveillance capitalism,’ a system that “unilaterally claims human experience as free raw material for translation into behavioural data.”^63^ This means that the more consumers engage with digital platforms, the more information is provided to TNCs to create unsolicited and personalised advertising that is highly effective in influencing consumer behaviour. This information is also used to increase target audience reach, ad memorability, and brand likeability.^64^

Finally, the acceptability and likeability of TNCs are crucial in the profitability of these corporations. Proactive CSR initiatives have been a powerful mechanism to create a stronger intent to purchase from the company,^65^ and the communication of CSR initiatives can increase positive public attitudes to unhealthy commodities and legitimise their consumption.^66^ This increases sales and creates a receptive environment to loosen or resist regulations, as these corporations obtain greater “social and reputational resources.”^67^ In emerging markets, CSR has been demonstrated as a valuable non-market strategy to “help reduce transaction costs when market-supporting institutions are absent or weak” whilst also increasing investment and future sales.^67^ Similarly, public-private partnerships (PPPs) also provide corporations with reputational benefits, even when PPPs tackling NCDs rarely result in positive outcomes.^68^ In fact, PPPs generally lead to government policies being ‘watered-down’ to the minimum level of intervention acceptable to industry, resulting in narrow policy responses and voluntary, rather than mandatory and enforceable, commitments.^69^

The challenge of tackling Big Food’s marketing practices is multi-faceted and complex, and thus any remedial action taken should be comprehensive and multisectoral. Otherwise, Big Food will switch to unregulated media and channels, as the tobacco industry did following the introduction of restrictions on cigarette marketing.^70,71^

####  Political Practices

Big Food directly impacts health through political strategies used to foster favourable policy and regulatory conditions for market expansion, and to sustain and protect its markets in the long-term. The ability of TNCs to undertake these political practices intensifies with the further concentration and consolidation of these already large TNCs.^72^

####  Capturing Policy – Fostering Favourable Regulatory Environments

Industry efforts to shape government policy in ways favourable to its commercial interests, known as corporate political activity, has been identified as a substantial challenge to NCD prevention efforts.^73^ This is because Big Food’s covert ‘below-the-line’ activity often leads to the implementation of ‘watered-down’ NCD prevention programmes, where Big Food’s profits are privileged over the health of the population.^74^ The expanding economic power associated with growing market shares of TNCs exacerbates their political power. The prevalence of such behaviours has led public health experts to propose that corporations producing unhealthy products should not be involved in the development of public health policies.^75^

Big Food uses a range of corporate political activities to ensure that implemented policy represents its interests. One key tactic used is lobbying, which is “any legal attempt by individuals or groups to influence government policy or action.”^76^ This typically involves TNCs hiring an external company to persuasively communicate their interests to a legislator or government official.^77,78^ Other tactics used alongside lobbying include direct and indirect financial incentives to political parties and policy-makers. Direct incentives take the form of donations, gifts, and other financial inducements, whilst indirect incentives include promises of economic benefits from employment, production, and supply of UPF. Another common tactic used to disincentivise governments from employing stricter, typically much more effective, public health policies is through the threat of legal action.^79,80^

Finally, Big Food influences policy through policy substitution. Whilst this can involve providing amended versions of policies that benefit the corporation or industry, it usually occurs through the introduction of ‘self-regulatory’ codes of conduct. The four MICs that have self-regulatory codes on advertising to children – South Africa, Mexico, Thailand and Brazil – are all countries where government regulation had been proposed.^81-83^ Research has shown that these codes have very low efficacy in changing industry behaviour as they are typically designed to replace government regulation without affecting sales.^84,85^

####  Capturing Science – Fostering Favourable Knowledge Environments

Big Food engages in evidence shaping to make governments disregard legitimate science.^18,86^ Tactics used include funding research that seeks to obscure public health evidence, disseminating data that favours industry, using unpublished evidence to obstruct policy, hosting scientific events,^87^ and criticising evidence to emphasise complexity or uncertainty.^88^ These strategies were used in China, where the industry-funded research organisation International Life Sciences Institute (ILSI) has facilitated industry involvement in ‘scientific’ research and events as well as having successfully lobbied the Chinese government to reframe its obesity policy.^27^ Chinese policy now argues that a lack of physical activity is the main causal factor for obesity, and that physical activity rather than diet should be the main focus for interventions.

This policy frame strongly contrasts with how public health organisations argue that obesity is a normal response to an obesogenic environment characterised by the ubiquitous marketing and availability of UPFs, and that the UPF industry should be regulated to reduce obesity.^89^ ILSI also appears to have been successful in shaping health policy in India.^90^

Big Food actively seeks to reduce the ability and credibility of public health organisations and researchers to advocate for regulation of the UPF industry. This includes threats to sue individual scientists and/or research institutions, monitoring individuals’ movements and using the media to launch character assassinations.^91,92^ Big Food also infiltrates and distracts the public health community by poaching advocates to work for industry-funded research groups, such as the Coca-Cola-funded Global Energy Balance Network.^93^ Working for such groups reduces the credibility of advocates that oppose industry tactics. Similarly, Big Food provides funding to public health organisations to stifle their ability to advocate for system reform. For example, Coca-Cola has funded programmes with the Mexican Federation of Diabetes and Funsalud, which subsequently stopped advocating for health system reform.^94,95^

####  Capturing Civil Society – Mobilising a Grassroots Lobby for Big Food

TNCs use PPPs, CSR, and sponsorship to generate a smokescreen of goodwill with civil society organisations, sports groups, and community members who can be called on to lobby for the corporation. This smokescreen is primarily driven by TNC investment in external research, services, and programmes where a powerful alliance of inter-dependent, co-opted organisations – including public relations agencies, management consulting firms, advertising agencies, and key media companies – are used to reshape how civil society perceives Big Food. These organisations help Big Food shift from defensive strategies that deny the role of its products in promoting NCDs to more conciliatory strategies that emphasise TNCs’ role in ostensible solutions to combatting such NCDs.^96^ Investments in CSR and PPP programmes, along with arguments about the economic value of TNCs, can also be used to co-opt some elements of civil society as ‘grassroots’ lobby groups that advocate for regulations favouring TNCs.^97^

###  Country Case Examples – South Africa, Colombia and Indonesia

 In this section, we examine the UPF and UPB sales and consumption patterns in South Africa, Colombia, and Indonesia, as well as market and political practices used by TNCs within these countries.

####  South Africa

####  Nutrition Status and UPF Sales Trends

Dramatic nutritional changes have occurred in South Africa in the last 20 years with the proportion of overweight girls increasing from 8.9% in 2000 to 29.4% in 2016 and obesity in children increasing from 1.8% in 2000 to 12.8% in 2016.^98^ Similar rises have occurred in adults.^98^ These changes occurred despite the South African Government having comprehensive policies that respond to the major NCD risk factors,^99^ including the establishment of a health promotion levy on SSBs in November 2017.

**Figure 3:**
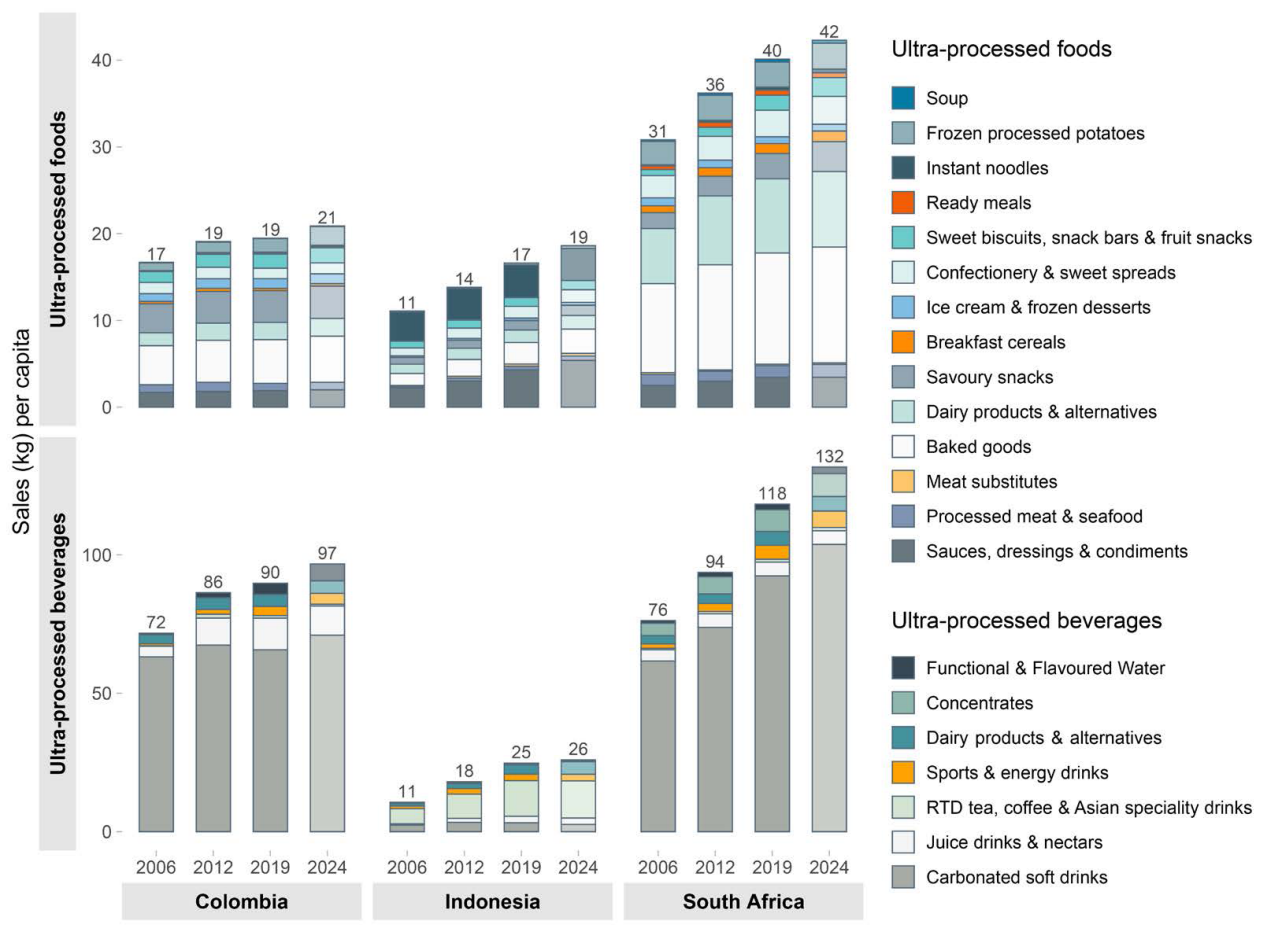
UPF and UPB Sales Per Capita in South Africa, Colombia, and Indonesia, 2006-2019 With Projections to 2024. Abbreviations: UPF, ultra-processed food; UPB, ultra-processed beverage.

The increases in overweight and obesity rates have been mirrored by high and increasing per capita UPF and UPB sales. As shown in Figure 3, per capita UPB sales increased by 55% between 2006 and 2019, with a further rise of 12% anticipated by 2024. Similarly, UPF sales per capita increased by 29% between 2006 and 2019. This was accompanied by high market concentration within SSBs.^100^

####  Market Practices

The significant growth of UPF and UPB products in South Africa has been accompanied by increased availability through supply chains and distribution systems, through the expansion of supermarkets into townships and vertically-integrated networks of informal vendors^101^; affordability^102^; and acceptability^100^ through changes to product design and increased marketing.^103,104^ Big Food has been highly active in implementing CSR initiatives, including physical activity and food distribution programmes, with the South African departments of Basic Education, Sport and Recreation, and Health and Agriculture.^105^ These include the Nestlé Healthier Kids Initiative which aims to provide Nestlé products to 50% of all South African primary school students in the guise of ‘nutrition,’^106^ and Coca-Cola’s youth employment program which sponsors the ownership of spaza shops in townships.^107^ Additionally, TNCs have moved to create “deep and broad market penetration linked to people’s passions, even into the townships and rural areas.”^108^ This has primarily been done by appealing to South Africans’ love of sport – for example, Coca-Cola’s sponsorship of the 2010 FIFA World Cup.^109^

####  Political Practices 

Big Food has used front groups, such as the ILSI, and trade associations, such as the Beverage Association of South Africa, to advocate for its interests. When an SSB tax was introduced in 2018, the Beverage Association and American Chamber of Commerce in South Africa publicly argued and lobbied the government that the introduction of such a tax would represent significant job losses that would destabilise the national economy.^105^ Their corporate submissions on the tax misrepresented evidence, in a way that did not “observe widely accepted approaches to the use of either scientific or economic evidence,”^110^ to argue that the tax would not improve health outcomes.

Following the proposal of government regulation for food advertising to children, Big Food in South Africa lobbied for a policy substitution. This led to the development of two voluntary codes for marketing to children, which focused on regulating television and school-based advertisements.^111,112^ Like most self-regulatory initiatives,^84^ these codes were ineffective in changing industry behaviour, with foods of low nutritional value accounting for 53% of all food advertising in peak after-school child viewing time in 2011,^113^ up from 50% in 2006.^114^

####  Colombia

####  Nutrition Status and UPF Sales Trends

Overweight and obesity rates increased in Colombia from 45.9% to 56.5% between 2005 and 2015; increases were observed in men and women across all ages, and in both rural and urban inhabitants.^115^ There has also been a steady increase in the prevalence of diabetes over the last 30 years in Colombia^116^ with 4000 people between 30 and 70 years old estimated to die prematurely every year from diseases related to obesity.^117^

Colombia is a growing market for value-added, processed, and packaged food products. It has a high level of per capita sales for UPBs and has seen considerable growth across both UPFs and UPBs, with per capita UPF sales projected to continue to grow over the next four years (Figure 3).

####  Market Practices

TNCs in Colombia have invested significantly in PPPs and CSRs, which may help explain why the Colombian government provides Big Food with significant leeway around regulations. For example, in 2016 the government recognised Postobón, the largest Colombian beverage company which provides significant financial support to five health foundations that operate major hospitals within Colombia,^118^ as one of Colombia’s most innovative corporations. This allowed Postobón to reduce how much it pays in income tax.^119^ Postobón is not alone in providing significant support to civil society organisations. Coca-Cola FEMSA, the franchise bottler for Coca-Cola in Latin America, also works closely with multilateral organisations, cultural institutions, governments, and civil society.^120^

####  Political Practices

Lobbying and coalition management have been core strategies used by Big Food to resist the implementation of NCD prevention policies in Colombia. For example, Postobón and its allies, including the National Association of Businessmen of Colombia, had over 90 lobbyists working to influence legislators during the soda tax bill debate. These lobbyists argued that the soda tax would reduce jobs and negatively affect the owners of independent stores and the economy.^117^ During committee hearings on the bill, in a blatant violation of the rules of the Colombian Congress, these lobbyists sat next to legislators.^117^ This bill did not pass despite widespread community support.

Finally, Big Food has sought to intimidate organisations who advocate for NCD prevention. For example, in 2016 Postobón filed a complaint against a commercial created by Educar Consumidores, a civil society organisation, with the government’s consumer protection agency. The commercial in question showed that consuming four sugary drinks a day equates to 47 teaspoons of sugar. Despite evidence supporting this claim, the agency ruled in favour of Postobón and ordered the advertisement to be withdrawn. After its withdrawal, Educar Consumidores employees reported that their phones and computers were hacked and placed under surveillance.^117^ The organisation’s director also reported being personally intimidated by threats made over the phone and in person.^117^

####  Indonesia

####  Nutrition Status and UPF Sales Trends

Over the last three decades, Indonesia has undergone a profound socioeconomic and epidemiological transition. Seven out of ten Indonesians now experience NCD-related deaths^122^ with dietary risks being one of the three leading factors of death.^123^ Between 2007 and 2018, overweight and obesity rates in Indonesian adults increased from 26.3% to 35.4%, with the percentage of obese individuals increasing from 10.5% to 21.8%.^124^

Between 1999 and 2014, Indonesians’ caloric intake of pre-prepared and packaged food nearly doubled.^125^ With the largest population in Southeast Asia and the fourth largest in the world, Indonesia represents potential for significant market growth for Big Food, particularly as UPB sales per capita have more than doubled since 2006.^126^

####  Market Strategies

To expand UPF markets within Indonesia, Big Food is making significant investments in advertising and marketing. Compared to the previous year, in 2016 the UPB industry increased its advertising spending by 33% to US$1.4 billion whilst the UPF industry increased its advertising spending by 54% to US$700 million.^127^

Given high levels of television viewing by Indonesian adults (around 4.3 hours per day)^128^ and children (around 7.4 hours per day),^129^ the marketing expenditure of Big Food is focused on television advertisements. Big Food has consistently ranked amongst the top three highest buyers of television advertising in Indonesia.^127,130,131^Its expenditure has focused on children, with 15 minutes of every hour of children’s television programming being food advertising.^132^

####  Political Practices

Big Food exercises great influence over the decisions of the Indonesian government as it is one of the highest contributors towards Indonesia’s gross domestic product outside of the oil and gas sector.^133^ Nestlé appears to have a close relationship with the Ministry of Industry, as the Minister remarked in 2019 that he hoped Nestlé would become an “investment ambassador of Indonesia in the food and beverage sector.”^134^ The importance of this working relationship, and the Ministry’s relationship with other TNCs, was recognised when the food and beverage industry was named as one of the five priority sectors in the Indonesian Government’s economic growth plan.^135^ Since the release of this plan, Nestlé has invested US$100 million to increase its manufacturing capacity in Indonesia.^134^

To generate further goodwill, and gain access to new markets, Big Food has undertaken CSR initiatives and engaged in PPPs in Indonesia. For example, Nestlé has established partnerships with schools and NGOs through its Nestlé Healthy Kids program^136^ and distributed 1.6 million food and beverage products during the coronavirus disease 2019 (COVID-19) pandemic.^137,138^ These practices are not isolated to Nestlé, with Coca-Cola Amatil Indonesia and Mondelez Indonesia also undertaking significant CSR projects to strengthen their relationships with the government, local NGOs, and religious institutions.^139,140^

Big Food has also sought to reframe public debates to ensure the continuation of its markets within Indonesia. For example, the Association of Indonesian Soft Drink Producers opposed the suggested introduction of an SSB tax by the Indonesian Finance Minister in 2020. The Association falsely claimed that such a tax will bring about no health benefits and result in the loss of 120000 jobs.^141,142^

###  Recommendations for Public Health Campaigns

The previous sections outline the considerable political and economic power of Big Food, the practices it uses to maintain this power, and the coterie of co-opted organisations that support them. These sophisticated practices can seem overwhelming to those seeking to limit the corporate power and health harms of Big Food. However, it is important to remember that tobacco control advocates felt similarly in the 1960s and 1970s.^143^ The major problem is one of *how* rather than *what*. We know what to do in terms of the strategies and programmes that need be implemented. The major barriers lie in how to garner the necessary political, bureaucratic, and civil society support to implement these effective public health controls.^144^ Drawing on the latest evidence on factors enabling the successful passage of policies and regulations targeting Big Food^145^ as well as the successes of the tobacco control movement, this section outlines some of the recommendations for public health campaigns.

###  Get the Right People 

The right people, with the right skills, training, and experience, are key to countering Big Food’s power, and reducing harms from UPFs. This includes bringing in people who have expertise in implementing and administrating public health interventions as well as countering industry attacks on these programmes. A study of the factors that supported the successful passage of the SSB tax in Mexico shows that good leadership, including skills in organisation, cooperation, planning and the ability to effectively partner with other sectors, is essential to the successful implementation of NCD prevention interventions.^146^ This was also evident in Thailand, where the Prime Minister’s Office was able to bring the right people together from education, agriculture, law enforcement, finance, transport, academia, and civil society to lead its NCD response.^147^ Based on local and international ideas and evidence, this team used its diverse skillsets to “put the interests of people before self and commercial interests.”^148^ This specific mix of skills helped the Thai government to introduce SSB, tobacco and alcohol taxes.

###  Build Networks to Pool Resources

Building networks of individuals and organisations with a shared purpose is an essential driver of political commitment and nutrition policy change.^149^ The importance of networks was evident in the passing of Mexico’s non-essential foods and a peso-per-litre tax on SSBs in 2013. This campaign involved activists bringing together 22 NGOs and 690 civil society organisations from public health and consumer rights perspectives to advocate for the tax’s implementation. With significant philanthropic financial support, this network was able to build relationships with legislators and undertake strategic communication campaigns and community outreach that were key to the bill’s passage.^74,146,150-152^

Networks need to be developed, expanded, nurtured, and supported over the longer-term^153^ as the formation, expansion and support of coalitions are crucial to resisting and overcoming the political power of Big Food. Whilst membership diversity helps build the credibility of a network, it also presents a significant challenge in terms of developing unified and effective responses. This emphasises the need for strong leadership, opportunities for ongoing dialogue, and the development of shared norms within a network.^149^ Lessons on successful networking can be drawn from transnational tobacco networks, that have brought together researchers, advocates, and international and national health organisations to embed tobacco control in HICs and, increasingly, MICs.^154^

Finally, public health networks cannot operate in isolation. In fact, they should learn from Big Food TNCs who, despite competing against each other in the marketplace, collaborate and pool organisational, financial, and human resources to undermine, delay, or stop effective public health action.^110,155^ To increase their credibility, networks should partner with practitioners and researchers from a broad range of disciplines, including international agencies, bilateral aid agencies, philanthropists,^156^ and journalists.^157^ Additionally, there are many aligned and genuinely non-conflicted, non-health harming organisations which work in trade, poverty alleviation, environment and education as well as in the private sector that public health could partner with. However, practitioners often do not sufficiently understand the perspectives or language of these sectors enough to effectively partner with them. As Bronwyn King, founder of Tobacco Free Portfolios, explains: “I needed to learn the language, systems, structure, rules and regulations that defined the whole finance sector… only then, when I understood the landscape from within, could I advocate for change” (Personal communication, 2020). Building these cross-sectoral alliances and drawing on the expertise of other aligned sectors and organisations will allow public health practitioners to build more comprehensive, effective, and ultimately successful campaigns for NCD prevention.^158^

###  Governments Need to Step Up

Similar to tobacco control,^154^ governments ought to be very cautious about working with highly conflicted UPF corporations.^159^ Governments, not just NGOs, should be monitoring the upstream drivers of harmful consumption of UPF and their levels of production, cost, availability, advertising, and sponsorship. Governments should also monitor and be transparent about political donations, major investors, funding of research, and the legislative and regulatory environment relevant to these products. This is more likely to occur with strong support from ‘cohesive, responsive and strongly led’ public health networks.^160,161^

###  Expand What ‘Counts’ as Public Health Skills 

To reduce the harmful impacts of Big Food, we need to actively recruit people who have the skillsets that are typically missing in public health teams. This should include bringing in people with lived experience of NCDs as well as digital strategists who understand how we can adapt and utilise the rapidly changing and expanding digital media ecosystem to advance health.^62,151^ We also need to work with business, trade, and governance analysts to help us develop and frame how we communicate our strategies to policy-makers and civil society.^146^ We need to build a cohort of political strategists who understand the political system and can work across government.^146^ We need investigative journalists who are passionate in uncovering the truth and standing up to corporate power.^157,161^ Finally, we need to find persuasive advocates and lawyers who are prepared to fight for people’s health.^146,161-163^

## Discussion

Across the globe, Big Food is becoming increasingly powerful and strategic in how it grows its market in MICs. Examples used in this article of Nestlé and Coca-Cola are the norm, not the exceptions. Whilst UPF sales in HICs are only marginally expanding, we have shown that the growth and sales of Big Food is rapidly expanding in MICs and that the combined sales of MICs currently outweigh sales in HICs (Figure 1). These sales are projected to grow significantly over the next decade as the lower per capita UPF and UPB consumption in heavily populated MICs increases at a much higher rate than HICs.

Our data shows how important MICs are for Big Food’s current and future growth. We are greatly concerned that the growth of Big Food has been exacerbated by the COVID-19 pandemic. COVID-19 is drawing public attention away from NCDs^166^ and TNCs are quickly adapting their market practices within MICs to maximise their penetration in this environment. Additionally, government funding for NCD prevention is likely to significantly decline during and after the pandemic, which will lead to increased calls for multi-stakeholder responses, typically PPPs and CSR, to prevent NCDs in MICs. This will allow highly opportunistic Big Food TNCs to further embed themselves within many MICs.

 However, public health cannot effectively counteract these profound long-term trends unless we understand how Big Food is growing and sustaining UPF markets within MICs. In this article, we first considered the market practices that TNCs use to advance their economic performance, exploring how they have established global production networks, created hyper-local distribution networks, and scaled up their digital marketing and involvement in CSR and PPP programmes. We then explored how TNCs are changing government policies, challenging scientific expertise, and co-opting civil society into a grassroots lobby. Government and practitioners need to understand these practices to create interventions that counteract the growth of Big Food.

 Although we use the term ‘transnational’ to describe Big Food corporations throughout this article, it would be more appropriate to refer to them to as ‘supranational corporations.’ This is because the size, power, global reach, and capacity of these corporations allow them to circumvent the laws and regulations of countries in which their products are produced and consumed in, effectively allowing them to operate ‘above’ the nation state. Interestingly, this is happening whilst TNCs become increasingly hyper-local in their market practices, which has also bolstered the development of domestic UPF corporations with transnational ambitions. The challenge of regulating these ‘supranational corporations,’ and their hyper-local interests is increasingly clear as national governments struggle to regulate the borderless digital ecosystem where digital marketing is increasingly being used by Big Food to target consumers within MICs.

Whilst the corporate political activities of Big Food corporations are increasingly studied and monitored,^78,94^ the academic study of these activities within MICs is relatively new.^97^ To counteract the expansion of Big Food, academic study “that investigates industrial diseases and the corporations that drive them”^7^ needs to expand and move from descriptive studies to studies that can underpin effective interventions. This includes interventions within the digital ecosystem, where public health practitioners need to understand the reach and impact of digital marketing by Big Food, and how Big Data informs their practices, in order to proactively work with civil society and policy-makers to set the parameters within which Big Food marketing can operate.

 Our study had several limitations. First, our perspective is limited by our positionality with only two authors being from MICs. Second, our study was not comprehensive as it did not include the experiences of policy-makers and public health advocates within MICs. Third, our analysis was primarily empirical with limited engagement with political theory. Fourth, we focused on high-level global trends, without analysing trends for UPF/UPB subcategories or regional data. Finally, the quality of our analysis relies on quality of the analysed market sales data. Future studies on this topic should fill the gaps left by this study by developing better data sets, engaging more heavily with political theory, exploring the nuances of UPF/UPB data, and including the lived experience of people in MICs.

## Conclusion

The country case examples of South Africa, Colombia, and Indonesia demonstrate how Big Food uses a combination of sophisticated market and political practices to maximise sales, minimise civil society and scientific opposition, and win over local politicians and bureaucrats.^26,28,36,78,167^ Yet, the NCD prevention policies of countries such as Thailand, Mexico, and South Africa demonstrate that governments and civil society can effectively curb the expansion of Big Food. Based on these experiences, we proposed recommendations for how public health campaigns can counter the influence of Big Food in MICs. We argue that this would involve developing an expanded global network of driven and passionate people with diverse skillsets, and increased government leadership. With these elements in place, we will then have better tools to be able to oppose the pernicious activities of Big Food and improve public health.

## Acknowledgements

 The authors would like to thank Kathryn Backholer, Trish Cotter and Piyali Somaia for their wise counsel.

## Ethical issues

 Ethical review was not required for the study given that it analyses secondary data. However, we recognise that there are two major ethical concerns regarding the equitable representation of research within this paper, particularly related to the three country case examples. First, there are no representative authors from South Africa and Colombia. Second, views from governments and/or ministries on TNC behaviours and/or initiatives were not included in this article as it was not intended to be exhaustive.

## Competing interests

 Authors declare that they have no competing interests.

## Authors’ contributions

 RM and EB conceived the paper and its design and wrote the first outline of the paper. PB provided extensive input that was used to restructure the paper. EJLK, LP and JW contributed to the country case examples. TMS and PB provided the quantitative data and graphs; JW and EJLK edited manuscript. All authors contributed to manuscript revision, read, and approved the submitted version.

## Disclaimers

 The views expressed in this article are those of the authors.

## Authors’ affiliations

^1^Melbourne School of Population and Global Health, University of Melbourne, Melbourne, VIC, Australia. ^2^College of Public Health, Medical and Veterinary Sciences, James Cook University, Townsville, QLD, Australia. ^3^International Center for Equity in Health, Federal University of Pelotas, Pelotas, Brazil. ^4^Indonesian Adolescent Health Association, Jakarta, Indonesia. ^5^Institute for Physical Activity and Nutrition, Deakin University, Geelong, VIC, Australia.
